# Hsa-miR-875-5p exerts tumor suppressor function through down-regulation of EGFR in colorectal carcinoma (CRC)

**DOI:** 10.18632/oncotarget.9944

**Published:** 2016-06-10

**Authors:** Tiening Zhang, Xun Cai, Qi Li, Peng Xue, Zhixiao Chen, Xiao Dong, Ying Xue

**Affiliations:** ^1^ Oncology Center, Shanghai General Hospital, Shanghai Jiaotong University, School of Medicine, Shanghai 200080, P. R. China

**Keywords:** hsa-miRNA-875-5p (miR-875-5p), EGFR, colorectal carcinoma (CRC), proliferation, apoptosis

## Abstract

Hsa-miRNA-875-5p (miR-875-5p) has recently been discovered to have anticancer efficacy in different organs. However, the role of miR-875-5p on colorectal carcinoma (CRC) is still ambiguous. In this study, we investigated the role of miR-875-5p on the development of CRC. The results indicated that miR-875-5p was significantly down-regulated in primary tumor tissues and very low levels were found in CRC cell lines. Ectopic expression of miR-875-5p in CRC cell lines significantly suppressed cell growth as evidenced by cell viability assay, colony formation assay and BrdU staining, through inhibition of cyclin D1, cyclin D2, CDK4 and up-regulation of p57(Kip2) and p21(Waf1/Cip1). In addition, miR-875-5p induced apoptosis, as indicated by concomitantly with up-regulation of key apoptosis protein cleaved caspase-3, and down-regulation of anti-apoptosis protein Bcl2. Moreover, miR-875-5p inhibited cellular migration and invasiveness through inhibition of matrix metalloproteinases (MMP)-7 and MMP-9. Further, oncogene *EGFR* was revealed to be a putative target of miR-875-5p, which was inversely correlated with miR-875-5p expression in CRC. Taken together, our results demonstrated that miR-875-5p played a pivotal role on CRC through inhibiting cell proliferation, migration, invasion, and promoting apoptosis by targeting oncogenic *EGFR*.

## INTRODUCTION

Colorectal carcinoma (CRC) is one of the leading causes of cancer mortality around the world, particularly in developed countries. If a patient is diagnosed at an advanced stage of CRC, his 5-year survival rate is only up to 10% [1, 2]. Clarification of the mechanisms that underlie CRC tumorigenesis and progression therefore is needed urgently [4, 5]. Though alterations in oncogenes and tumour suppressor genes have been reported in CRC [6–8], the precise molecular mechanisms underlying CRC pathogenesis remain to be fully elucidated. MicroRNAs (miRNAs) are a class of small, highly conserved, and non-coding RNAs that directly target genes' 3**'**-untranslated regions (3**'**-UTRs) by binding to some sequence-specific sites, resulting in deceased expression of these genes [9–12]. Numerous of publications have confirmed that dysregulation of miRNAs play an important role in various types of cancers [9–16]. Selective miRNA expression contributes to tumor proliferation, apoptosis, senescence, cell identity, stem cell maintenance and metastasis [9–21]. While there are still numerous of unknown details about the role of miRNAs on human cancers that still need to be investigated [22].

MiR-875-5p (MIMAT0004922), is down-regulated in numerous of diseases, including neurodegenerative diseases [22], and cervical cancer cells [23]. It is also reported that miR-875-5p targets the HPV genomic sites, and negatively influences exogenous and endogenous E6 gene expression. Additionally, high level of miR-875 inhibits cell growth and promotes apoptosis in SiHa cells [23]. These results suggest tumor-suppressive functions of miR-875-5p in cancer but up to now this suggestion has not been rigorously tested.

Over-expression and activation of EGFR plays a positive role on cell growth and metastasis in variety of solid tumors including CRC [24–27]. EGFR tyrosine kinase activation leads to activation of numerous of intracellular signals, which bring to an end in processes that are critical to tumor progression, including cell growth, epithelial-mesenchymal transition (EMT), metastasis, and angiogenesis. These changes are mediated by numerous of downstream targets of EGFR, including extracellular signal-regulated kinase 1/2 (ERK1/2) and AKT protein kinase [28, 29]. Although EGFR signaling pathway is crucial and is well studied in CRC progression, how miR-875-5p mediated EGFR signaling to modulate CRC progression is little known.

The goal for our current study is to explore the biological functions of miR-875-5p on CRC and to investigate the underlying mechanisms of action. We show for the first time that miR-875-5p directly targets and regulates the 3**'**-UTR of the human EGFR (NM_005228) mRNA, which is up-regulated in many cancers, including CRC. Here, we reported that miR-875-5p is indeed suppressed in primary CRC compared with the matching adjacent normal colorectal tissues, and found 3**'**-UTR of the human EGFR mRNA is really a target of miR-875-5p. Collectively, we discovered that miR-875-5p suppressed cell proliferation, metastasis, and promoted cell apoptosis by directly targeting 3′-UTR of *EGFR* in CRC.

## RESULTS

### MiR-875-5p is down-regulated in human CRC tissues and cell lines, and benefits for prognosis

**Figure 1:**
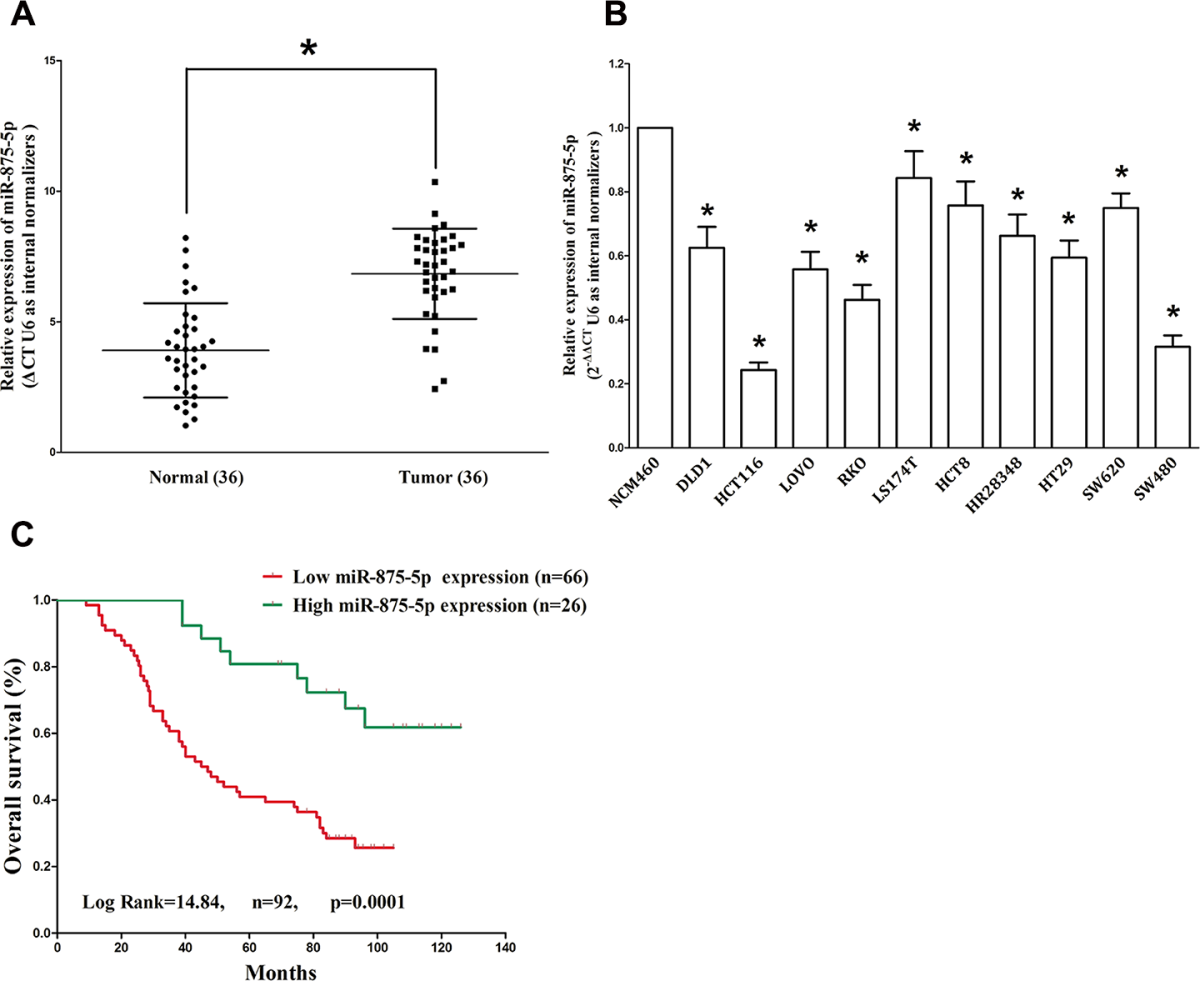
MiR-875-5p is down-regulated in primary human CRC and CRC cell lines, and benefits for prognosis (**A**) miR-875-5p is significantly decreased in primary human CRC tissues in comparison to adjacent-normal CRC tissues. *n* = 36 for each group. (**B**) The expression level of miR-875-5p in six CRC cell lines and normal NCM460 cells. Assays were performed in triplicate. (**C**) Kaplan-Meier survival analysis revealed that down-regulated miR-875-5p is associated with poor prognosis in patients with colorectal carcinoma. *P < 0.001, Means ± SEM was shown. Statistical analysis was conducted using student *t*-test and Log Rank test.

To determine whether miR-875-5p is decreased expression in CRC, we measured miR-875-5p expression in human primary CRC and pair-matched adjacent colorectal normal tissues by qRT-PCR. We used U6 that is not deregulated in CRC for normalization. Results demonstrated miR-875-5p expression in the tumors was significantly (*P* < 0.05) reduced (mean = 32% of decrease) in 92 CRC cancers in comparison to their matched controls among 92 samples analyzed (Figure 1A). Next, we examined miR-875-5p expression in CRC cell lines, and results demonstrated a lower expression of miR-875-5p in HCT116, LOVO, RKO, LS174T, HCT8, HR28348, SW480, SW620, DLD-1 and HT29 cell lines, compared with that of in normal colorectal epithelial cells, NCM460 (Figure 1B). Among the ten CRC cell lines, miR-875-5p decreased the most in HCT116 and SW480 cell lines, thus, we chose HCT116 and SW480 for model of CRC cell lines. In addition, to assess the clinical significance of miR-875-5p, we evaluated the association between its expression with clinic-pathological parameters (i.e., stage, maximum diameter and lymph node metastasis). Results demonstrated miR-875-5p expression levels in CRC patients were significantly corrected with tumor size (*P* = 0.0057), differentiation (*P* = 0.0007), TNM stage (*P* = 0.0005), and lymph node metastasis (*P* = 0.0048). However, miR-875-5p expression was not associated with other clinical characteristics such as age (*P* = 0.7452), gender *(P* = 0.4916) or Tumor site (*P* = 0.2393) in CRC patients (Table 1). Additionally, Kaplan–Meier survival analysis demonstrated that CRC patients with low miR-875-5p expression levels (≤ 32% of decrease, *n* = 66) of had shorter overall survival, in comparison to patients with high miR-875-5p expression levels (> 32% of decrease, *n* = 26) (Figure 1C), which demonstrated decreased expression of miR-875-5p was associated with poor prognosis. Thus, down-regulated expression of miR-875-5p might play a crucial role on CRC progression and development.

**Table 1:** Correlation between miR-875-5p expression and clinicopathological parameters of CRC patients (*n* = 92)

Parameter	*n*	Relative miR-875-5p expression		
		Low	High	*P*-value [a]
Age/years				0.7452
≤ 50	40	28	12	
> 50	52	38	14	
Gender				0.4916
Male	55	38	17	
Female	37	28	9	
Differentiation				0.0007 [*]
Well, moderate	60	50	10	
Poor	32	16	16	
Tumor size (cm in diameter)				0.0057 [*]
≤ 5 cm	36	20	16	
> 5 cm	56	46	10	
Lymph node metastasis				0.0048 [*]
Positive	53	32	21	
Negative	39	34	5	
TMN stage				0.0005 [*]
I	23	10	13	
II/III/IV	69	56	13	
Tumor site				0.2393
Proximal colon	30	19	11	
Distal colon	21	14	7	
Rectum	41	33	8	

a Chi-square test

* *P* < 0.05

### Expression of EGFR is up-regulated in primary human CRC and negatively expressed related to miR-875-5p

**Figure 2:**
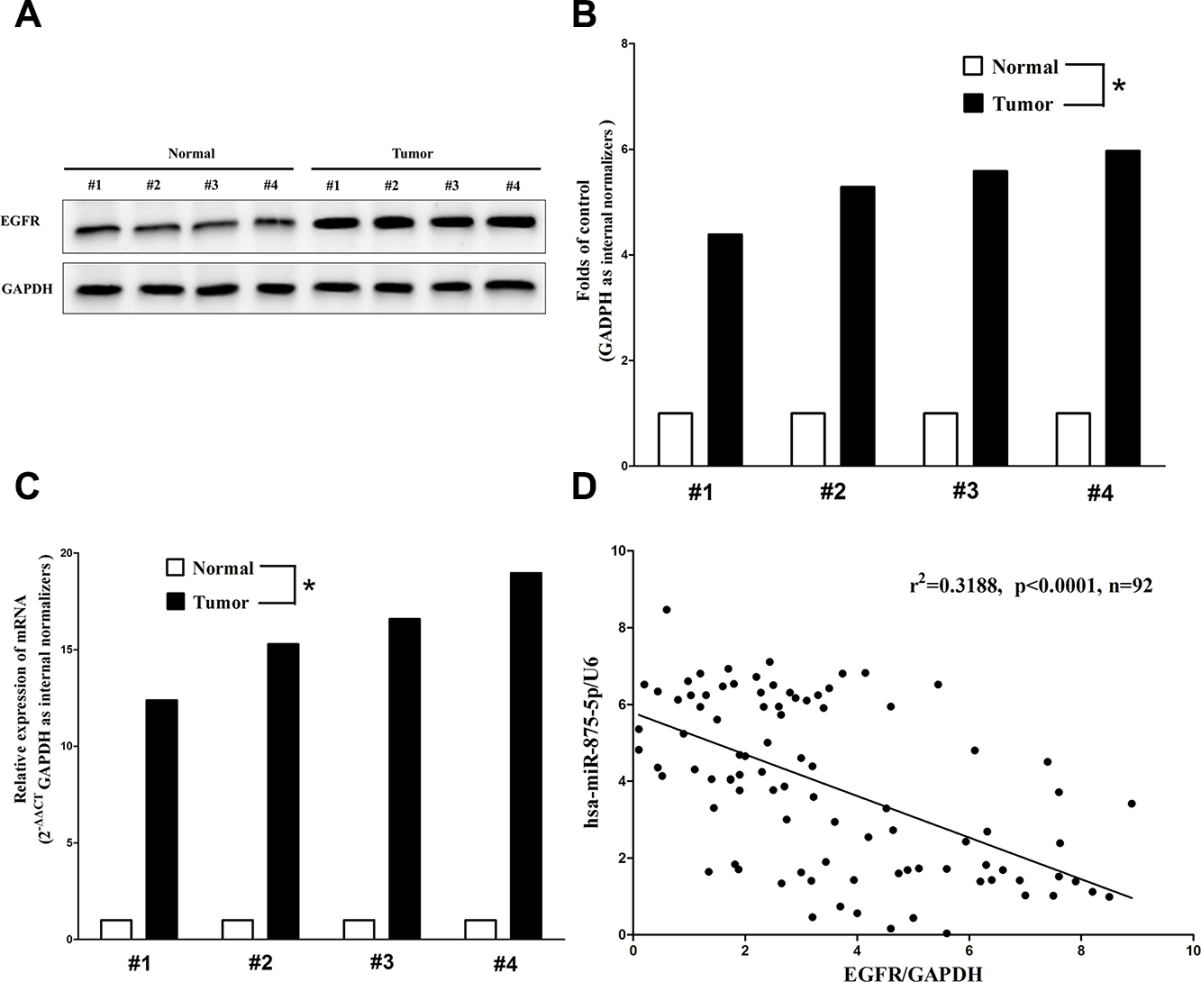
Expression of *EGFR* is up-regulated in primary human CRC and negatively expressed related to miR-875-5p (**A–C**) Western-blot of EGFR protein and qRT-PCR of EGFR mRNA in CRC tissues and adjacent-normal CRC. *n* = 36 for each group. (**D**) Scatter plots showing the inverse association between miR-875-5p level and EGFR mRNA expression. *P < 0.001, Means ± SEM was shown. Statistical analysis was conducted using student *t*-test and person's correlation analysis.

EGFR is important oncogene that shown strong power of oncogenicity, by promotion of cell growth, migration, invasion and epithelial mesenchymal transition (EMT), as well as inhibition of cell apoptosis in many tumors including CRC [24, 29]. Thus, we next examined EGFR expression in human primary CRC and pair-matched adjacent colorectal tissues, and our western blot results demonstrated that EGFR protein was increased in CRC tissues compared with normal colorectal tissues (4.4-fold of increase) (Figure 2A). These results were confirmed by qRT-PCR of EGFR mRNA expression (Figure 2A). Since EGFR is the key role on regulation of cell cycle, aberrations of these three proteins might contribute to human CRC. Moreover, we estimated the association between EGFR mRNA levels and miR-875-5p levels in 92 CRC tissues. Results demonstrated expression levels of EGFR mRNA and miR-875-5p revealed a significantly negative correlation as the results of Pearson correlation analysis (r^2^ = 0.3188, *P* < 0.0001) (Figure 2B).

### MiR-875-5p targets human *EGFR*

**Figure 3:**
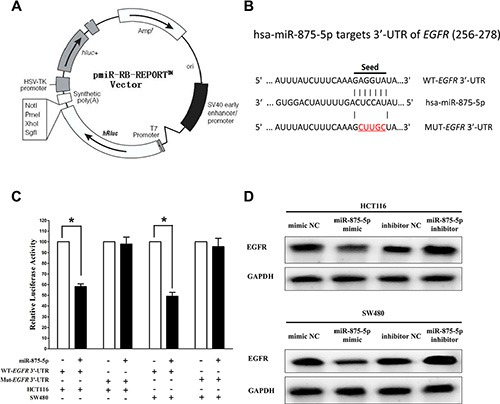
*EGFR* proto-oncogene is a target of miR-875-5p at specific 3′-UTR sites (**A**) pmiR-RB-REPORT TM dual-luciferase reporter vector. (**B**) The 3′-UTR of *EGFR* harbors one miR-875-5p cognate site. (**C**) Relative luciferase activity of reporter plasmids carrying wild-type or mutant *EGFR* 3′-UTR in HCT116 and SW480 cells co-transfected with negative control (NC) or miR-875-5p mimic. (**D**) Protein expression of EGFR in HCT116 and SW480 cells after transfected with related miRNAs. Assays were performed in triplicate. *P < 0.001, Means ± SEM was shown. Statistical analysis was conducted using student *t*-test.

We then investigated the potential molecular mechanism of the anti-tumorigenic property of miR-875-5p in CRC cells. Since miRNAs primarily exert their biological functions in animal cells by hampering the expression of target mRNA, we searched different data bases (TargetScan; microRNA.org and PicTar) for its potential targets that exhibited oncogenic properties. *EGFR*, which harbors one conserved miR-875-5p cognate site, namely, 256–278 of *EGFR* 3**'**-UTR) (Figure 3B), is a predicted target of miR-875-5p. Next, we used luciferase reporter assays to determine whether *EGFR* expression are indeed regulated by miR-875-5p, And results demonstrate that miR-875-5p inhibits luciferase activity by around 46% in HCT116 cells and 51% in SW480 cells when the reporter plasmid carried the WT *EGFR* 3**'**-UTR (Figure 3C), but no significant inhibition was observed at the reporter plasmid carried a mutant *EGFR* 3**'**-UTR. We next examined the role of miR-875-5p on the protein expression of EGFR. Our results of western blot demonstrated that miR-875-5p inhibited expression of EGFR protein by approximately 65% and 75%, when compared with blank HCT116 and SW480 cells (Figure 3D), respectively. Our results reveal that miR-875-5p targets human EGFR by directly binding to the predicted sites in 3**'**-UTR of EGFR mRNA.

### Inhibition of miR-875-5p does not reverse the anticancer efficacy of silence of *EGFR* expression *in vitro*

**Figure 4:**
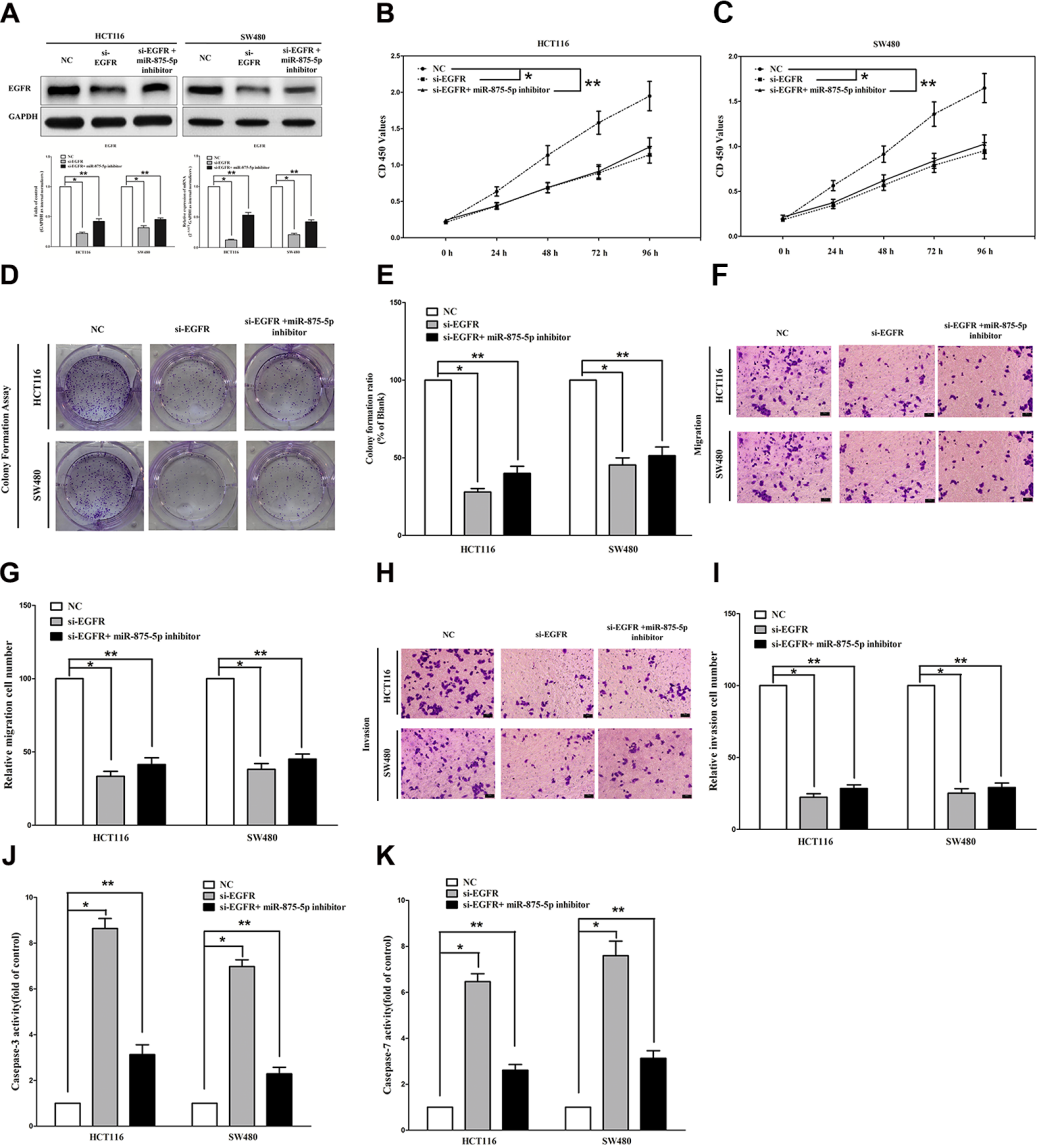
Inhibition of miR-875-5p does not reverse the anticancer efficacy of silence of EGFR expression *in vitro* (**A**) Western-blot of EGFR protein and qRT-PCR of EGFR mRNA in si-EGFR treated and blank HCT116 and SW480 cells, and si-EGFR *+* miR-875-5p inhibitor. (**B**–**C**) CCK8 assays of HCT116 and SW480 cells after transfected (un-transfected) with si-EGFR and si-EGFR *+* miR-875-5p inhibitor. (**D**–**E**) Shown are representative photomicrographs of colony formation assay after transfected with (without) si-EGFR and si-EGFR *+* miR-875-5p inhibitor for fourteen days. (**F**–**G**) Shown are representative photomicrographs of transwell migration assay after transfected with (without) si-EGFR and si-EGFR *+* miR-875-5p inhibitor. (**H**–**I**) Shown are representative photomicrographs of transwell invasion assay after transfected with (without) si-EGFR and si-EGFR *+* miR-875-5p inhibitor. (**G**–**K**) Quantitative representation of caspase-3 and caspase-7 activity in HCT116 and SW480 cells transfected with (without) si-EGFR and si-EGFR *+* miR-875-5p inhibitor for forty eight hours. Assays were performed in triplicate. *P < 0.001, **P < 0.001, Means ± SEM was shown. Statistical analysis was conducted using ANOVA.

We next examined the potential tumorigenicity of *EGFR* in CRC. Silence of *EGFR* expression by si-EGFR significantly inhibited the expression of *EGFR* (Figure 4A). Moreover, loss of *EGFR* expression also contributed to inhibition of CRC cell (both HCT116 and SW480 cells) growth (65% or 52% of decrease in HCT116 or SW480 cells) (Figure 4B–4E) and metastasis (63% or 68% of decrease in migration, 76% or 73% of decrease in invasion in HCT116 or SW480 cells) (Figure 4F–4I). In addition, inhibition of *EGFR* expression promoted apoptosis in CRC cell (8.5-fold of increase or 7.7-fold of increase at caspase 3 activity, 6.5-fold of increase or 7.8-fold of increase at caspase 7 activity in HCT116 or SW480 cells) (Figure 4J–4K). These results further verified the powerful tumorigenicity of *EGFR* in CRC. Thus, we adopted *EGFR* for as targeted oncogenes. However, inhibition of miR-875-5p does not reverse the anticancer efficacy of silence of *EGFR* expression in CRC cell lines (both HCT116 and SW480 cells). These results indicate the anticancer efficacy of miR-875-5p is partly attributed to its inhibitory role on *EGFR*.

### MiR-875-5p suppresses tumor growth nude mouse xenograft model

**Figure 5:**
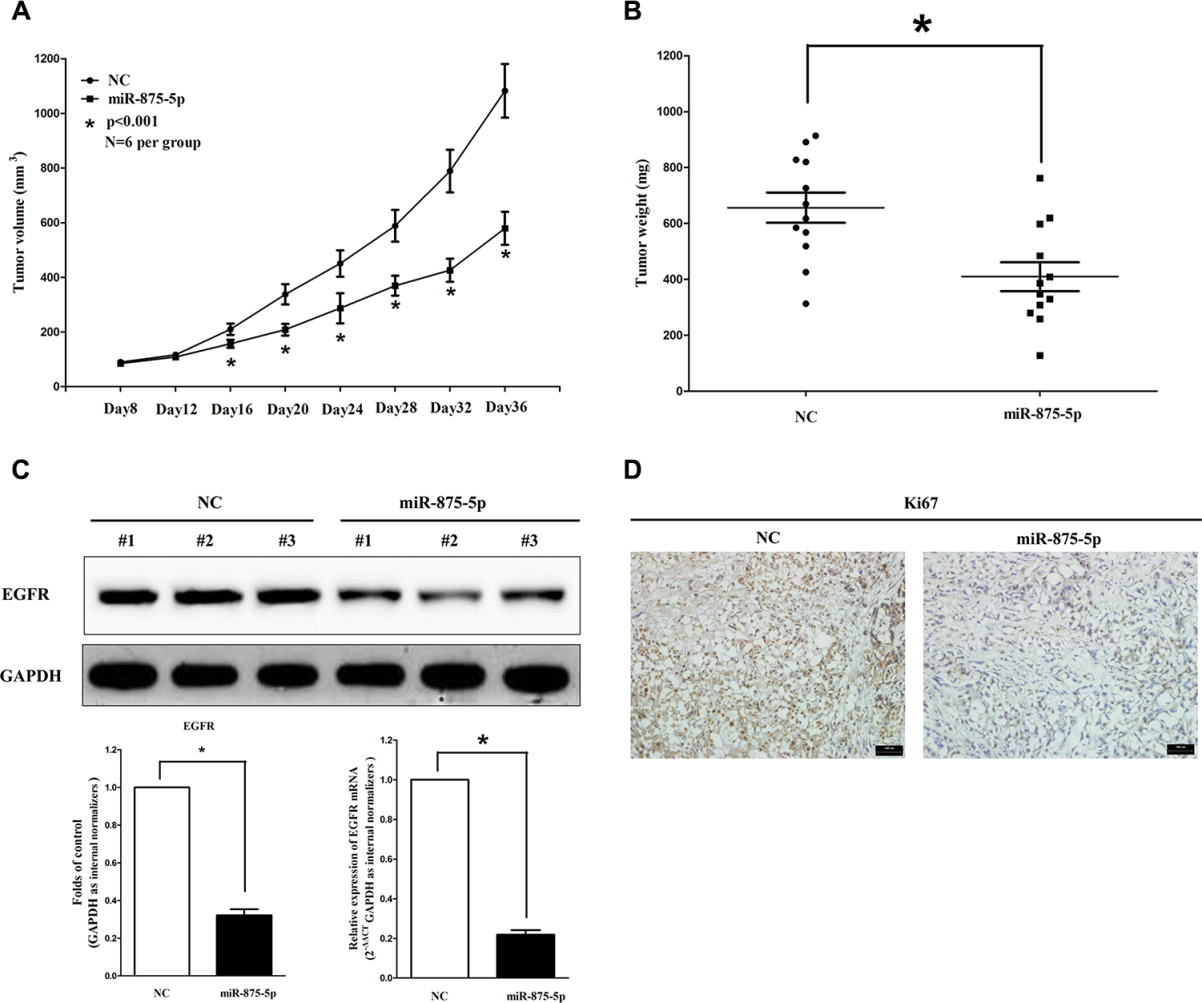
Ectopic expression of miR-875-5p suppresses tumor growth *in vivo* (**A**) Tumor volume in nude mice. (**B**) Tumor weight in nude mice. Each group contained six mice (*n* = 6); the data are presented as the mean ± SEM; *p < 0.001, compared with the NC group. (**C**) The expression of EGFR protein and mRNA in nude mice. Assays were performed in triplicate. *P < 0.001, Means ± SEM are shown. Statistical analysis was conducted using student *t*-test. (**D)** Immunohistochemistry showed miR-875-5p decreased the proliferation index Ki67.

To validate the tumor suppressive efficiency of miR-875-5p *in vivo*, we established a BALB/c nude mouse xenograft model using HCT116 cells. The mice were treated as descript in method part. Our results demonstrated that the tumor volume and weight of mice treated with miR-875-5p mimic were significantly reduced (55% of decrease in tumor weight) relative to that of treated with miR mimic NC (Figure 5A and 5B). This result demonstrates miR-875-5p significantly suppresses the tumorigenicity of HCT116 cells in the nude mouse xenograft model. In addition, our results of western-blot and qRT-PCR demonstrated that the decreased expression (67% of decrease) of EGFR in tumors developed from miR-875-5p-mimic-treated nude mice relative to that of control tumors (Figure 5C). Moreover, immunohistochemical staining of resected tumor tissues found that tumors formed from miR-875-5p-transfected HCT116 cells exhibited reduced positivity (78% of decrease) for Ki67 compared with those formed from control cells (Figure 5D). Thus, miR-875-5p reduces the growth of established colorectal carcinoma xenografts.

### MiR-875-5p inhibits CRC cell proliferation and colony formation

**Figure 6:**
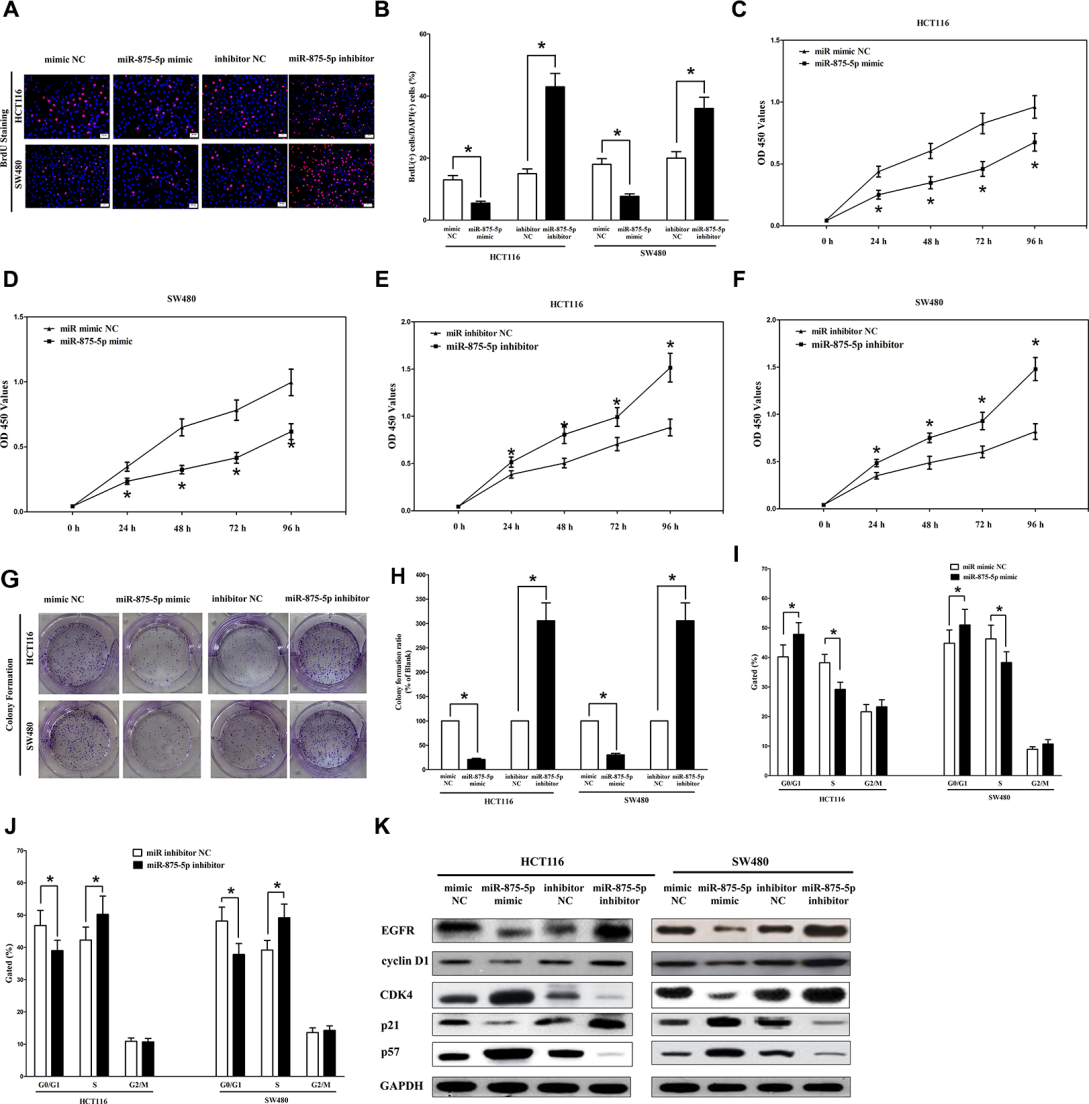
Ectopic expression of miR-875-5p inhibits proliferation and colony formation of HCT116 and SW480 cells (**A**) Shown are representative photomicrographs of BrdU staining after transfected HCT116 and SW480 cells with miR-875-5p mimic, miR-875-5p mimic NC, miR-875-5p inhibitor or miR-875-5p inhibitor NC for twenty four hours. Bar = 100 μm. (**B**) Statistical analysis of BrdU staining. (**C–F**) CCK8 assays of HCT116 and SW480 cells after transfected with miR-875-5p mimic, miR-875-5p mimic NC, miR-875-5p inhibitor, miR-875-5p inhibitor NC. (**G**) Shown are representative photomicrographs of colony formation assay after transfected with miR-875-5p mimic, miR-875-5p mimic NC, miR-875-5p inhibitor or miR-875-5p inhibitor NC for fourteen days. (**H**) Statistical analysis of colony formation assay. Assays were performed in triplicate. (**I–J)** Cell-cycle analysis was performed forty eight hours following the treatment HCT116 and SW480 cells with miR-875-5p mimic or miR-875-5p mimic NC, miR-875-5p inhibitor or miR-875-5p inhibitor NC. The DNA content was quantified by flow cytometric analysis. (**K**) Expression of cyclin D1, cyclin D1, CDK4, p21 and p57 protein in transfected HCT116 and SW480 cells. Assays were performed in triplicate. *P < 0.001, Means ± SEM was shown. Statistical analysis was conducted using student *t*-test.

To further explore its anticancer efficacy on CRC cell, we examined the role of miR-875-5p on CRC cell (HCT116 and SW480) proliferation. Our results of BrdU staining revealed that miR-875-5p inhibited HCT116 and SW480 cell DNA synthesis by approximately 60% (Figure 6A and 6B) and 53% (Figure 6A and 6B), compared with blank HCT116 and SW480 cells, respectively. However, miR-875-5p inhibitor treatment increased HCT116 and SW480 cell DNA synthesis by approximately 2.4 folds (Figure 6A and 6B) and 1.6 folds (Figure 6A and 6B) compared with blank HCT116 and SW480 cells, separately. To verify these results, we also did the CCK8 assay, and results demonstrated that miR-875-5p over-expression significantly attenuated HCT116 and SW480 cells vitality, while loss of miR-875-5p promoted cell proliferation (Figure 6C–6F). In addition, we also investigated the role of miR-875-5p on clonogenic survival, and results demonstrated miR-875-5p mimic treatment caused a decrease in the clonogenic survival of HCT116 and SW480 cells compared with blank HCT116and blank SW480 cells (Figure 6G and 6H), while miR-875-5p inhibitor-treated HCT116 cells showed an significant increase in the clonogenic survival, when compared with blank HCT116 and blank SW480 cells (Figure 6G and 6H). Furthermore, the growth-inhibitory role of miR-875-5p in HCT116 and SW480 cell lines was accompanied by a corresponding decrease in the proportion of cells in the S phase and an increase in the proportion of cells in G1 (Figure 6I and 6J).

We next examined the efficiency of miR-875-5p on expression of EGFR. Our results discovered miR-875-5p significantly inhibited the protein expression of EGFR, while loss of miR-875-5p remarkably increased the level of EGFR in HCT116 and SW480 cells (Figure 6K). cyclin D2 is highly expressed and promotes tumorigenesis in numerous tumors [30, 31]. In our research, the protein expression of cyclin D2 was repressed by over-expression of miR-875-5p (Figure 6K). Over-expression of CDK4 has been discovered in numerous of malignant neoplasms, including breast cancer, glioma, and CRC [32]. In our research, the protein expression of CDK4 was repressed by over-expression of miR-875-5p in HCT116 and SW480 cells (Figure 6K). Our study revealed over-expression of miR-875-5p is a mechanism for the up-regulation of p57 (a cyclin-dependent kinase inhibitor) level in CRC cell lines (HCT116 and SW480) (Figure 6K). Transfection of p21 (a cell cycle inhibitor) expressive constructs into normal [36] and tumor cell lines [37] leads to cell cycle arrest in G1 [38]. Our study revealed that miR-875-5p up-regulated p21 level in CRC cell lines (HCT116 and SW480) (Figure 6K).

It is concluded that miR-875-5p markedly inhibited cell growth in CRC cell lines.

### MiR-875-5p inhibits CRC cell metastasis

Invasion and migration through the basement membrane are characteristics of metastatic cancer cells. Then, we explored the role of miR-875-5p on HCT116 and SW480 cells migration and invasion.

**Figure 7:**
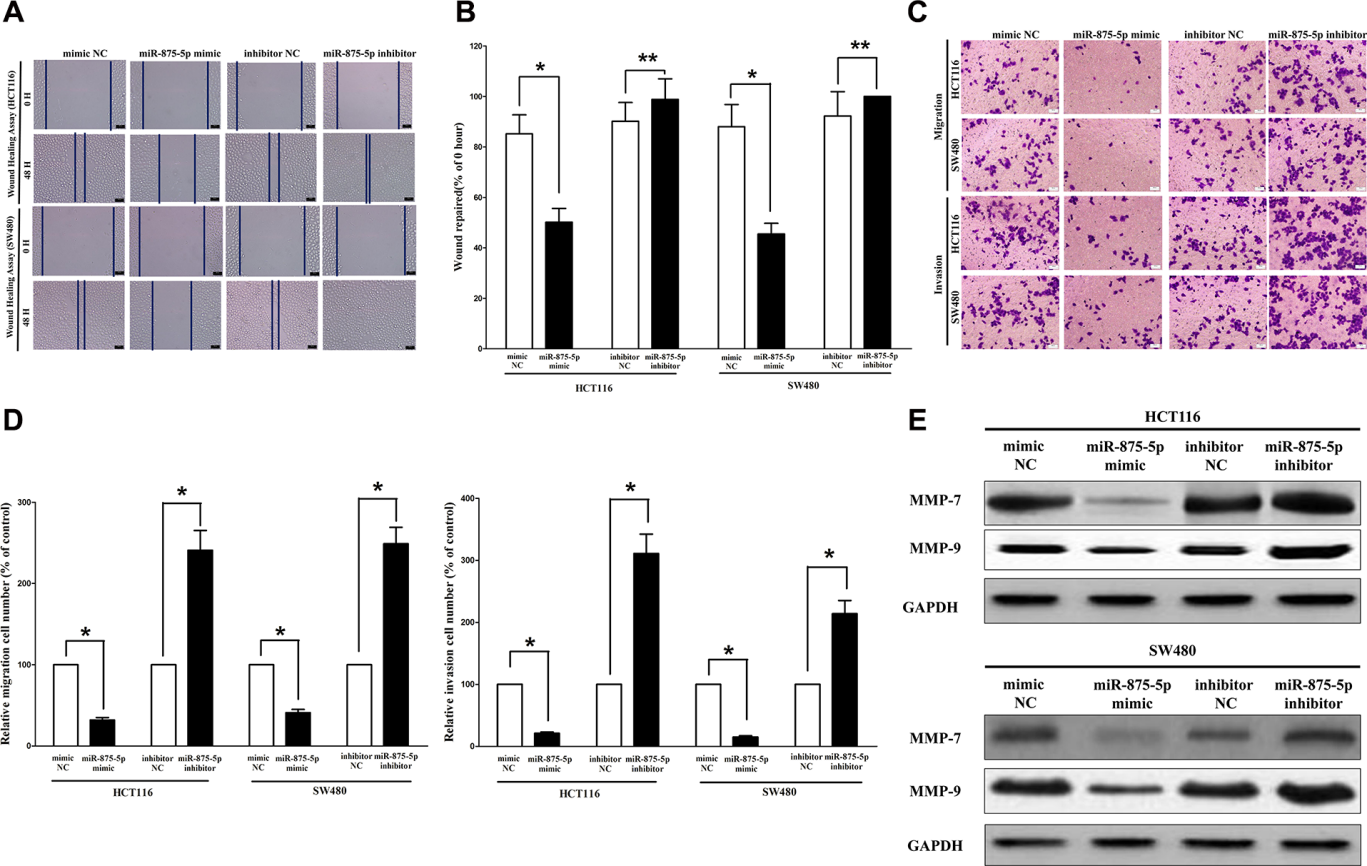
Ectopic expression of miR-875-5p in HCT116 and SW480 cells reduces cell migration and invasion motility (**A**) Shown are representative photomicrographs of “wound healing assay” in HCT116 and SW480 cells after transfected miRNAs for 0 hour and forty eight hours. Bar = 100 μm. (**B**) Statistical analysis of “wound healing assay”. (**C**) HCT116 and SW480 cells were loaded onto the top well of a transwell inserts for cell migration or invasion assay. After twenty four hours, cells that migrated to the bottom chamber containing serum-supplemented medium were stained with 0.1% crystal violet, visualized under a phase-contrast microscope, and photographed. Bar = 100 μm. (**D**) Total number of cells in five fields was counted manually. (**E**) Expression of MMP-7 and MMP-9 protein in HCT116 and SW480 cells after transfection. Assays were performed in triplicate. *P < 0.001, Means ± SEM was shown. Statistical analysis was conducted using student *t*-test.

We used two different approaches to assess the role of miR-875-5p on HCT116 and SW480 cells migration. In the first technique, we used a “scratch wound healing” assay. Motility of cells at different time points after generation of the wound was monitored under a microscope. Closure of the wound was complete within forty eight hours in control HCT116 and SW480 cells (Figure 7A and 7B). In contrast, miR-875-5p-expressing cells migrated to the wound at a much slower (60% of distance or 52% of distance in HCT116 or SW480 cells) rate (Figure 7A and 7B). In the second approach, we used transwell/migration assay to assess the role of miR-875-5p on cell migration. As expected, migration of miR-875-5p-expressing clones was inhibited by 65% in HCT116 and 56% in SW480 cells, compared with the blank HCT116 and SW480 cells (Figure 7C and 7D), respectively. However, when treated with miR-875-5p inhibitor, migration in miR-875-5p-expression defect HCT116 and SW480 cells were significantly increased by approximately 2.5 and 2.8 folds relative to blank HCT116 and SW480 cells (Figure 7C and 7D) respectively.

To investigate the role of miR-875-5p on HCT116 and SW480 cells invasion, we used a transwell invasion assay. As expected, invasion of miR-875-5p-expressing clones was inhibited by 68% in HCT116 and 77% in SW480 cells, relative to blank HCT116 and SW480 cells (Figure 7C and 7D), respectively. However, when treated with miR-875-5p inhibitor, invasion in miR-875-5p-expression defect HCT116 and SW480 cells were significantly increased by approximately 3.0 and 2.3 folds relative to blank HCT116 and SW480 cells (Figure 7C and 7D), separately.

We also investigated the role of miR-875-5p on expression of MMP-7 and MMP-9, which all play a key role on tumor metastasis, and results indicated miR-875-5p inhibited the protein expression of MMP-7 and MMP-9 both in HCT116 and SW480 cells (Figure 7E). As expected, loss of miR-875-5p significantly increased the protein expression of MMP-7 and MMP-9 in both HCT116 and SW480 cells (Figure 7E).

Taken together, these results clearly demonstrated that miR-875-5p expression markedly reduces the migration and invasion motility of CRC cells.

### MiR-875-5p promotes CRC cell apoptosis

**Figure 8:**
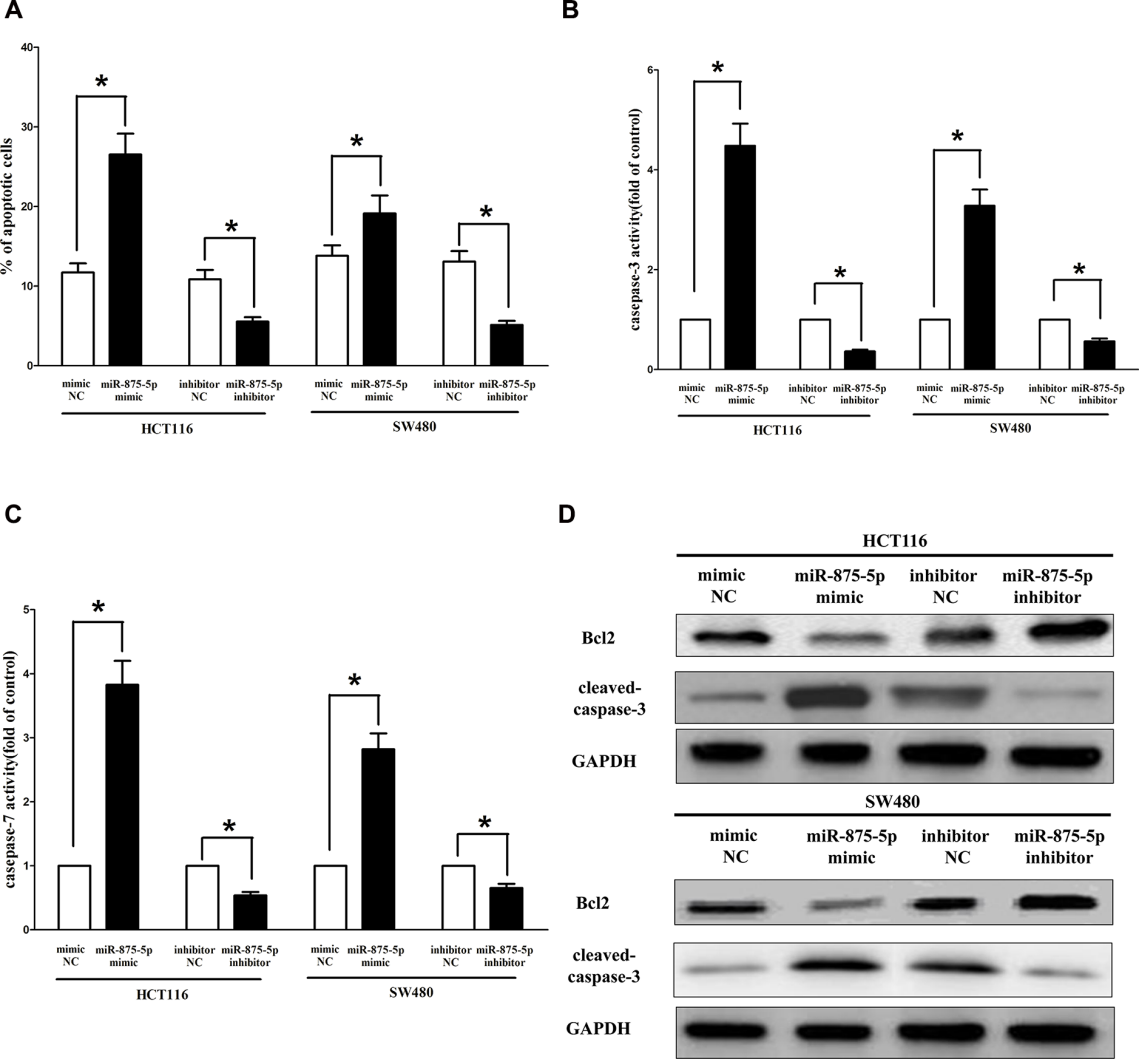
Ectopic expression of miR-875-5p promotes apoptosis in HCT116 and SW480 cells (**A**) Shown are statistical analysis of flow cytometric analysis. (**B–C**) Quantitative representation of caspase-3 and caspase-7 activity in HCT116 and SW480 cells transfected with related miRNAs for forty eight hours. (**D**) Western-blot of Bcl2 protein in HCT116 and SW480 cells after transfection. Assays were performed in triplicate. *P < 0.001, Means ± SEM was shown. Statistical analysis was conducted using student *t*-test.

Next, we examined the role of miR-875-5p on HCT116 and SW480 cells apoptosis. Our results of flow cytometric analysis (FCA) revealed that forced expression of miR-875-5p resulted in a ~2.3 folds and ~1.7 folds of increase in apoptotic cell death of HCT116 and SW480 cells (Figure 8A), respectively. However, the percentage of apoptotic cells induced by miR-875-5p was decreased to the basal level when the cells were treated with the specific miR-875-5p inhibitor (Figure 8A). In addition, we also tested the caspase-3 and caspase-7 activity after treatment of HCT116 and SW480 cells with relative RNAs, and results demonstrated that miR-875-5p remarkably increased caspase-3 and caspase-7 activities in HCT116 and SW480 cell lysate, by approximately 3.9 and 4.1 folds increase (caspase-3 activity), 2.8 and 3.3 folds increase (caspase-7 activity), than that of in bank HCT116 and SW480 cells (Figure 8B and 8C), respectively. However, loss of miR-875-5p by transfecting with miR-875-5p inhibitor remarkably reduced the caspase-3 and caspase-7 activity in HCT116 and SW480 cell lysate, compared with that of in bank HCT116 and blank SW480 cells (Figure 8B and 8C), respectively. Moreover, miR-875-5p also inhibited the expression level of anti-apoptotic protein Bcl2 (Figure 8D), and increased the protein expression of cleaved-caspase-3 (Figure 8D) in HCT116 and SW480 cells. These results demonstrated that miR-875-5p indeed promoted apoptosis in HCT116 and SW480 cells.

## DISCUSSION

Our present study has revealed the following novel findings: (i) exogenously overexpressed miR-875-5p suppresses tumor regeneration in 6 CRC xenograft models and suppresses cell growth *in vitro* and *in vivo*; (ii) miR-875-5p overexpression inhibits CRC cells metastasis; (iii) inhibition of miR-875-5p in CRC cells results in high clonal clonogenic, and tumorigenic properties; (iv) miR-875-5p overexpression promotes CRC cells apoptosis, and inhibition of miR-875-5p inhibits CRC cell apoptosis; (v) miR-875-5p targets *EGFR* in CRC cells and negatively expressed with *EGFR*.

Up to date, the molecular mechanisms about CRC progression are still sparsely elaborated. Hence, a better understanding of the molecular mechanisms referred to tumor formation and development will be helpful to exploit novel therapeutic strategies and targets for treatment of human CRC. Although dysregulation of miRNAs was reported in numerous of human cancers [39], aberrant expression and potential role of miRNAs in CRC were under studied. Our results also indicated that miR-875-5p was decreased in CRC tissues and cell lines, indicating disorder of miR-875-5p is an early event of CRC tumorigenesis.

We therefore explored the speculative tumor suppressive efficiency of miR-875-5p on human CRC cell lines. Firstly, we examined the mechanism of miR-875-5p on CRC cell growth, and results indicated restoration of miR-875-5p in the HCT116 and SW480 cells significantly suppressed cell growth as evidenced by colony formation assays, BrdU, and cell viability (CCK8). The growth-inhibitory role of miR-875-5p may be attributed to miR-875-5p targets 3**'**-UTR of EGFR mRNA, and inhibits the expression of EGFR in CRC cells. In addition, miR-875-5p also inhibited cyclin D1, cyclin D2 and promoted p57 and p21 expression levels in CRC cells, which further contributed to the growth-delay efficacy of miR-875-5p. In addition to inhibition of cell growth, its growth-inhibitory role was also associated with its promotion on cell apoptosis. We discovered that miR-875-5p induced cell apoptosis happens by the regulation of extrinsic apoptosis pathway, which had been viewed as a crucial antitumor mechanism [40–42]. The expression of critical anti-apoptosis protein Bcl2 was decreased after transfecting with miR-875-5p, and the activity of its downstream factor, active apoptosis executor caspase-3 was up-regulated, leading to initiate a caspase cascade, and finally resulted in cell apoptosis [43].

Restoration of miR-875-5p suppressed the cell metastasis *in vitro* assays. The reduced disseminating efficiency and cell motility induced by miR-875-5p in CRC cell lines were demonstrated to be related to decreased protein expression of cell migration and invasion molecules matrix metalloproteinases 7 (MMP-7) and matrix metalloproteinases (MMP-9). MMP-7 and MMP-9 are members of the matrix metalloproteinases (MMPs) family, which are located in extracellular and control basic cellular processes, such as morpho-genesis, migration and survival, and they can degrade extracellular matrix during the cancer metastatic process [43, 44]. MMP-7 is an assured rabble-rouser of aggressive behavior in numerous of cancers including CRC. MMP-9 is established as a crucial module for initiating of the pre-metastatic niche [43]. Thus, decreased expression of MMP-7 and MMP-9 by miR-875-5p led to alleviated cell metastasis ability.

Having shown the critical effects of miR-875-5p on suppressing CRC progression, we explored the potential gene effectors involving in its function. Amazingly, a single miRNA could affect numerous of target genes coincidently [43], and among the predicted target genes of miR-875-5p, we discovered EGFR acted as a crucial effector of miR-875-5p. Our results demonstrated miR-875-5p could significantly inhibit the luciferase activity of wide type of Luc-*EGFR*-3′-UTR by directly binding with the targeted sites of the 3′-UTR in EGFR mRNA. Therefore we selected EGFR as the target gene, and focused on it for further analysis.

In our present study, we discovered miR-875-5p was an underlying prognostic factor for CRC, and found miR-875-5p is remarkably decreased in human CRC tissues in comparison to normal colorectal tissues. Moreover, we also revealed over-expression of miR-875-5p inhibits cell growth, metastasis, and promotes cell apoptosis in CRC cell lines, through directly targeting EGFR. The present results elucidate a potential mechanism underlying the tumor-suppressor role of miR-875-5p, and indicate that miR-875-5p could be a useful marker and potential therapeutic target in colorectal cancer.

## MATERIALS AND METHODS

### Tissue collection

Fresh and formalin-fixed, paraffin-embedded, CRC tumor tissue samples were obtained from patients who were diagnosed with primary CRC. Elective surgery was carried out on these patients at Shanghai General Hospital of Shanghai Jiaotong University (Shanghai, China). In total, 36 pairs of fresh CRC and adjacent non-tumor tissues (more than 5 cm away from the tumor) were freshly frozen in liquid nitrogen and stored at −80°C until further use. 92 cases of archived, formalin-fixed, paraffin-embedded CRC tissue samples were collected and used in clinicopathological and prognostic investigation of miR-875-5p. A comprehensive set of clinicopathological data were recorded, including age, gender, size of primary tumor, tumor differentiation, T stage, lymph node metastasis, and distant metastasis. The stage of disease was determined according to the tumor size, lymph node, and metastasis (pTNM) classification system. The use of tissues for this study has been approved by the ethics committee of Shanghai General Hospital of Shanghai Jiaotong University. Before using these clinical materials for research purposes, all the patients have signed the informed consent. None of these patients received any pre-operative chemotherapy or radiotherapy.

### Cell Culture and transfection

Ten CRC cell lines (DLD1, HCT116, LOVO, RKO, LS174T, HCT8, HR28348, HT29, SW620, and SW480) and the NCM460 cell lines were purchased from the Institute of Biochemistry and Cell Biology of the Chinese Academy of Sciences (Shanghai, China). Cells were cultured in RPMI 1640 or DMEM (Gibco, Grand Island, NY, USA) medium supplemented with 10% fetal bovine serum (10% FBS), 100 U/ml penicillin, and 100 mg/ml streptomycin (Gibco) in humidified air at 37°C with 5% CO2. Hsa-miR-875-5p mimic and mimic negative control, hsa-miR-875-5p inhibitor and inhibitor negative control were purchased from GenePharma Co.,Ltd. (Shanghai, China). For convenience, hsa-miR-875-5p mimic and mimic negative control, hsa-miR-875-5p inhibitor and inhibitor negative control were simply referred to as miR-875-5p mimic and miR mimic NC, miR-875-5p inhibitor and miR inhibitor NC, respectively. Complete medium without antibiotics was used to culture the cells at least twenty-four hours prior to transfection. The cells were washed with 1× PBS (pH7.4) and then transiently transfected with 50 nM miR-875-5p mimic or miR mimic NC, 100 nM miR-875-5p inhibitor or miR inhibitor NC, using Lipofectamine™ 2000 (Invitrogen, Carlsbad, CA, USA) according to the manufacturer's instructions.

### Protein extraction and western blotting

Cells were rinsed twice with cold PBS and lysed by RIPA buffer (Thermo Fisher Scientific, Waltham, MA, USA) containing protease inhibitor cocktail (Roche). Protein (40 μg per sample) was separated by SDS-PAGE using a 10% polyacrylamide gel. The proteins were transferred electrophoretically onto a PVDF membrane. Blotted membranes were blocked in 5% skimmed milk diluted in TBST, followed by incubation with appropriate primary antibodies (anti-EGFR, anti-cyclin D1, CDK4, anti-cyclin D2, anti-p21, anti-p57, anti-MMP-7, anti-MMP-9, anti-cleaved caspase 3, and anti-GADPH; obtained from Cell Signaling Technology and all the antibodies were diluted 1:1000.) overnight at 4°C. The membranes were then washed for 5 minutes for three times with TBST, and subsequently incubated for 1 hour with HRP-linked secondary antibody (Cell Signaling Technology) at room temperature. GADPH was used as an internal control. The blots were detected using an enhanced chemiluminescence kit (Millipore) and subjected to autoradiography using X-ray film.

### RNA isolation and quantitative reverse transcription poly-merase chain reaction (qRT-PCR)

Total RNA from the cultured cells was extracted using Trizol reagent (Invitrogen) according to the manufacturer's instructions. MiRNA levels were measured by qRT-PCR. For the qRT-PCR detection of mature miR-875-5p expression, we purchased the Bulge-Loop™ miRNA qRT-PCR Primer Set and the miRNA qRT-PCR Control Primer Set (both from RiboBio). RNA (2 μg) was converted into cDNA using the PrimeScript™ RT reagent kit with gDNA Eraser (Takara, Dalian, China) according to the manufacturer's instructions. qRT-PCR was performed using SYBR^®^ Premix Ex Taq™ II (Takara) in the ABI PRISM^®^ 7300 real-time PCR system (Applied Biosystems, Foster City, CA,USA). GADPH and U6 were used as endogenous controls. In addition, melting curves were used to evaluate non-specific amplification. The relative expression level was calculated using the 2^−ΔΔCt^ method. The primer sequences used in this study are as follows: the primers of miR-875-5p were purchased from RiboBio (RiboBio Co., Ltd, Guangzhou, China); human EGFR: sense: 5**'**-CGAATGGGCCTAAGATCCCG-3**'**, antisense: 5**'**-GGAGCCCAGCACTTTGATCT-3′; human MMP-7: sense: 5**'**-GAGTGCCAGATGTTGCAGAA-3**'**, antisense: 5**'**-AAATGCAGGGGGATCTCTTT-3′; human MMP-9: sense: 5**'**-CTGCAGTGCCCTGAGGACTA-3**'**, antisense: 5**'**-ACTCCTCCCTTTCCTCCAGA-3**'**; The formula and its derivations were obtained from the ABI Prism 7300 sequence detection system user guide. Statistical analysis was performed on the fold change.

### Colony formation assay

Cells were transfected with miR-875-5p mimic or miR mimic NC, miR-875-5p inhibitor or miR inhibitor NC, as described above. Twenty-four hours later, transfected cells were trypsinized, counted and replated at a density of 1000 cells/6 cm dish. The medium was changed every three days. After ten days, the cells were washed with PBS, fixed with 4% paraformaldehyde for 30 minutes, and then stained with crystal violet for 30 minutes for visualization and counting. Colonies containing at least 50 cells were scored. Each assay was performed in triplicates.

### Luciferase reporter assays

The 3**'**-untranslated region (UTR) of human *EGFR* was amplified from human genomic DNA and individually inserted into the pmiR-RB-REPORT TM (Ribobio, Guangzhou, China) using the XhoI and NotI sites. Similarly, the fragment of *EGFR* 3**'**-UTR mutant was inserted into the pmiR-RB-REPORT TM control vector at the same sites. For reporter assays, HCT116 cells were co-transfected with wild-type (mutant) reporter plasmid and miR-875-5p mimics (miR mimic NC) using Lipofectamine 2000 (Invitrogen). Firefly and Renilla luciferase activities were measured in cell lysates using the Dual-Luciferase Reporter Assay system. Luciferase activity was measured forty-eight hours post-transfection using dual-glo luciferase reporter system according to the manufacturer's instructions (Promega, Madison, WI, USA). Firefly luciferase units were normalized against Renilla luciferase units to control for transfection efficiency.

### Transwell migration/invasion assay

HCT116 and SW480 cells were grown in RPMI 1640 containing 10% fetal bovine serum to ~60% confluence and transfected with 50 nM miR-875-5p mimic or a negative control, 100 nM miR-875-5p inhibitor or a negative control. After twenty-four hours, the cells were harvested by trypsinization and washed once with Hanks' balanced salt solution (Invitrogen). To measure cell migration, 8-mm pore size culture inserts (Transwell; Costar, High Wycombe, UK) were placed into the wells of 24-well culture plates, separating the upper and the lower chambers. In the lower chamber, 500 μL of RPMI 1640 containing 10% FBS was added. Then, serum-free medium containing 5 × 10^4^ cells were added to the upper chamber for migration assays, whereas 1 × 10^5^ cells were used for matrigel invasion assays. After twenty-four hours of incubation at 37°C with 5% CO_2_, the number of cells that had migrated through the pores was quantified by counting 10 independent visual fields under the microscope (Olympus) using a ×20 magnifications, and cell morphology was observed by staining with 0.1% crystal violet. Filters were washed thoroughly with 1× PBS and dissolved in 500 μL of 33% acetic acid, and absorbance was measured at 570 nm. Absorbance of cells incubated in the serum-free medium in the bottom chamber was used as negative control. Each experiment was performed at least three times.

### BrdU immunofluorescence assay

HCT116 and SW480 cells were seeded on sterile cover glasses placed in the 6-well plates. After transfection with miR-875-5p mimic, miR mimic NC, miR-875-5p inhibitor, miR inhibitor NC for forty eight hours, the BrdU (5-bromo-2-deoxyuridine; Sigma) stock solution at 10 mg/mL in saline was diluted 1000× in the culture medium and incubated for 60 min. After washing with 1× PBS, cells were then fixed for 20 min in 4% paraformaldehyde (PFA) and permeabilized with 0.3% Triton X-100 for 10 min. After blocking with 10% goat serum in 1× PBS for 1 h, cells were incubated with a primary rabbit antibody against BrdU (1:200, Abcam) over night at 4°C, and then incubated with the secondary antibody coupled to a fluorescent marker, Cy3, at room temperature for 2 h. After DAPI staining and 1× PBS washing, the cover slips were mounted on to glass slides with anti-fade solution and visualized using a fluorescence microscope (Olympus 600 auto-biochemical analyzer, Tokyo, Japan) with Image-Pro Plus software for image analysis, and 10 microscopic fields were taken for calculating BrdU.

### CCK8 assay

Cell growth was measured using the cell proliferation reagent WST-8 (Roche Biochemicals, Mannheim, Germany). After plating cells in 96-well microtiter plates (Corning Costar, Corning, NY) at 1.0 × 10^3^/well, 10 μL of CCK8 was added to each well at the time of harvest, according to the manufacturer's instructions. One hour after adding CCK8, cellular viability was determined by measuring the absorbance of the converted dye at 450 nm.

### Tumor formation in BALB/c nude mice

BALB/c athymic nude mice (male, 4–6-weeks old and 16–20 g) were purchased from Hubei Research Center of Laboratory Animal (Wuhan, China). All animal experiments were carried out in accordance with the Guide for the Care and Use of Laboratory Animals of Wuhan University. To establish CRC xenograft model, 5 × 10^5^ HCT116 cells were suspended in 100 μL phosphate-buffered saline and inoculated subcutaneously into the flanks of nude mice. After 8 days, the transplanted nude mice were randomly divided into two groups (*n* = 6 each). miR-875-5p mimic (miR-875-5p) or miR mimic NC (NC) (RiboBio Co., Ltd, Guangzhou, China) was directly injected into the implanted tumor at the dose of 1 nmol (in 20 μL phosphate-buffered saline) per mouse every 4 days for seven times. The tumor size was monitored by measuring the length (L) and width (W) with calipers every 4 day, and the volumes were calculated using the formula: (L × W^2^)/2. Mice were killed by cervical dislocation in day 28, and the tumors were excised and snap-frozen for protein and RNA extraction.

### Immunohistochemistry

Immunohistochemistry of the tumor tissues was performed as described previously [44–48]. 3-μm tumor sections were incubated with commercial rabbit polyclonal antibodies against Ki67 (Affinity) at 1/100 dilution overnight at 4°C. Then, the sections were conjugated with horseradish peroxidase (HRP) antibody (1:500 dilution; Santa Cruz Biotechnology, Santa Cruz, CA) at room temperature for 2 h, then covered by DAB (Vector Laboratories, Burlingame, CA), and slides were mounted with Vectashield mounting medium (Vector Laboratories). Subsequently, all fields were observed under light microscopy (Olympus 600 auto-biochemical analyzer, Tokyo, Japan). Control experiments without primary antibody demonstrated that the signals observed were specific.

### Flow cytometry

#### Apoptosis analysis

HCT116 and SW480 cells transfected with miR-875-5p mimic or negative control were trypsinized and resuspended in 1× binding buffer at 1 × 10^6^ cells/mL. 100 μL of this cell suspension was incubated with 5 μL of FITC-Annexin V and 5 μL propridium iodide (PI) for 15 minutes in the dark. The reaction was terminated with the addition of 400 μL 1× binding buffer and analyzed with FACSCalibur using the CellQuest software (Becton Dickinson). FITC-Annexin V-positive and PI-negative cells were considered as apoptotic and the experiments were carried out in triplicates.

### Cell-cycle analysis

Transfected cells were harvested forty-eight hours after transfection. The cells were fixed in 70% ethanol, washed once with PBS, and then labeled with propidium iodide (Sigma-Aldrich) in the presence of RNase A (Sigma-Aldrich) for 30 min in the dark (50 g/mL). Samples were run on a FACSalibur flow cytometer (Becton-Dickinson, FL, NJ, USA), and the percentages of cells within each phase of the cell cycle were analyzed using Cell Quest software.

### Wound healing assay *in vitro*

The HCT116 and SW480 cells were seeded in 6-well plates and incubated for twenty-four hours. Then a linear wound was tehncreated by dragging a 100-μL pipette tip through the monolayer prior to transfection. Cellular debris was removed by gentle washes with culture medium, following which transfection was performed immediately, and the cells were allowed to migrate for a further forty-eight hours. The healing process was dynamically photographed after the wound was introduced using a microscope (Olympus 600 auto-biochemical analyzer, Tokyo, Japan). Migration distance was measured from images (5 fields) taken at each indicated time point. The gap size was analyzed using Image-Pro Plus 6.0 software. The residual gap between the migrating cells from the opposing wound edge was expressed as a percentage of the initial gap size.

### Caspase-3/7 activity assay

The activity of caspase-3/7 was determined using the caspase-3/7 activity kit (Beyotime Institute of Biotechnology, Haimen, China). To evaluate the activity of caspase-3/7, cell lysates were prepared after their respective treatment with various designated treatments. Assays were performed on 96-well microtitre plates by incubating 10 μL protein of cell lysate per sample in 80 μL reaction buffer (1% NP-40, 20 mM Tris-HCl (pH 7.5), 137 mM Nad and 10% glycerol) containing 10 μL caspase-3 substrate (Ac-DEVD-pNA) (2 mM). Lysates were incubated at 37°C for 4 h. Samples were measured with an ELISA reader at an absorbance of 405 nm. The detail analysis procedure was described in the manufacturer's protocol.

### Statistical analysis

All experiments were repeated 3 times independently. The results are presented as the means ± standard error mean (SEM). Two independent sample *t*-test or One-Way Analysis of Variance (ANOVA) was performed using SPSS 19.0 software in order to detect significant differences in measured variables among groups. A value of *P* < 0.05 was considered to indicate a statistically significant difference.

## Abbreviations

The abbreviations used are: used: miR: microRNA
miR-875-5p: has-microRNA-875-5p
colorectal carcinoma: CRC
3′-untranslated region: 3′-UTR
B-cell lymphoma-2: BCL2.
